# Spatio-temporal dynamics and drivers of highly pathogenic avian influenza H5N1 in Chile

**DOI:** 10.3389/fvets.2024.1387040

**Published:** 2024-05-02

**Authors:** Claudio Azat, Mario Alvarado-Rybak, José F. Aguilera, Julio A. Benavides

**Affiliations:** ^1^Sustainability Research Center & PhD in Conservation Medicine, Life Science Faculty, Universidad Andrés Bello, Santiago, Chile; ^2^Núcleo de Investigaciones Aplicadas en Ciencias Veterinarias y Agronómicas, Facultad de Medicina Veterinaria y Agronomía, Universidad de las Américas, Santiago, Chile; ^3^MIVEGEC, Institut de Recherche pour le Développement, CNRS, Université de Montpellier, Montpellier, France

**Keywords:** bird flu, epidemiology, HPAI, one health, pelicans, poultry

## Abstract

**Introduction:**

Highly pathogenic avian influenza A H5N1 clade 2.3.4.4b (hereafter H5N1) is causing vast impacts on biodiversity and poultry around the globe. In Chile, lethal H5N1 cases have been reported in a wide range of wild bird species, marine mammals, backyard and industrial poultry, and humans. This study describes the spatio-temporal patterns of the current epizootic of H5N1 in Chile and test drivers that could be associated with outbreak occurrence.

**Methods:**

We used H5N1 cases reported by the Chilean National Animal Health Authority from 5 December 2022 to 5 April 2023. These included wild bird cases confirmed through an avian influenza-specific real-time reverse transcription PCR assay (RT-qPCR), obtained from passive and active surveillance. Data were analyzed to detect the presence of H5N1 clusters under space–time permutation probability modeling, the association of H5N1 with distance and days since the first outbreak through linear regression, and the correlation of H5N1 presence with a number of ecological and anthropogenic variables using general linear modeling.

**Results:**

From 445 H5N1 identified outbreaks involving 613 individual cases in wild birds, a consistent wave-like spread of H5N1 from north to south was identified, which may help predict hotspots of outbreak risk. For instance, seven statistically significant clusters were identified in central and northern Chile, where poultry production and wildlife mortality are concentrated. The presence of outbreaks was correlated with landscape-scale variables, notably temperature range, bird richness, and human footprint.

**Discussion:**

In less than a year, H5N1 has been associated with the unusual mortality of >100,000 individuals of wild animals in Chile, mainly coastal birds and marine mammals. It is urgent that scientists, the poultry sector, local communities, and national health authorities co-design and implement science-based measures from a One Health perspective to avoid further H5N1 spillover from wildlife to domestic animals and humans, including rapid removal and proper disposal of wild dead animals and the closure of public areas (e.g., beaches) reporting high wildlife mortalities.

## 1 Introduction

The current panzootic of highly pathogenic avian influenza A subtype H5N1 clade 2.3.4.4b (hereafter H5N1) is causing widespread mortality among wild birds and mammals worldwide (1–3). In addition, more than half a billion poultry have died or been culled as a control measure (4), threatening the food security of low- and middle-income countries. First described in the Netherlands in October 2020, the novel H5N1 virus bearing the clade 2.3.4.4b HA gene (5) originated from reassorted avian influenza H1N1, H3N8, and H5N8 viruses (5–7). This novel H5N1 virus has spread rapidly through major avian migratory pathways to every continent except Australia (7–10). Recently, evidence of H5N1 infection associated with mortality in wild birds and mammals has confirmed its arrival in Antarctica (11, 12). Although it has major impacts on wild and domestic birds, the unusually high and growing number of lethal cases among marine and terrestrial mammals, especially in Europe and the Americas, is a hallmark of the current emergency (2, 13–16). Unprecedented mammal-to-mammal transmission has been increasingly suspected. For instance, Agüero et al. (17) described the mortality of several thousand farmed American minks (*Neogale vison*) in northern Spain following a rapid pen-to-pen pattern of spread. Furthermore, spillover has affected people, with 10 cases of H5N1 human infections reported since January 2022, two of them fatal, fueling fears of the emergence of a new global pandemic with unknown consequences (18, 19).

In Chile, the Agriculture and Livestock Service (SAG) reported the first case of H5N1 in a Peruvian pelican (*Pelecanus thagus*) found dead on 5 December 2022, in the northern coastal city of Arica. This case was confirmed using a specific real-time reverse transcription PCR assay (hereafter RT-qPCR), as described by Ariyama et al. (20). This occurred a few weeks after Peru reported mass mortalities of >22,000 marine birds (mainly *P. thagus*), presumably due to avian influenza (1). Two months later, Chile reported the first case of backyard poultry in the coastal town of Chañaral de Aceituno, followed by the first confirmed cases in marine mammals in a South American sea lion (*Otaria flavescens*) and a marine otter (*Lontra felina*), also in the North of the country. Subsequently, the first outbreak of industrial poultry in Central Chile was reported in March 2023 (in the city of Rancagua), where the country’s poultry production is concentrated. Later, on 29 March, the Ministry of Health reported Chile’s first human case of H5N1 in a 53-year-old man from the coastal city of Tocopilla in northern Chile, raising the possibility of this panzootic becoming a serious threat to public health.

Despite multiple non-pathogenic and highly pathogenic avian influenza viruses circulating across the globe for decades and previous Influenza A pandemics, including H1N1 Spanish flu (1918) and H1N1 swine flu (2009), there is still a lack of understanding of the factors contributing to the wide and rapid spread of the novel H5N1 virus, particularly given its high mortality and diverse range of infected hosts. A better understanding of the ecology of H5N1 is urgently needed to inform effective strategies for disease surveillance and control in Chile and elsewhere. This study describes the spatio-temporal patterns of the current epizootic of H5N1 in Chile and tests ecological and anthropogenic drivers that may be associated with outbreak occurrence to help predict and prevent areas of high risk for future outbreaks in wildlife, domestic poultry, and potential cases in humans.

## 2 Materials and methods

### 2.1 Virus surveillance

We used cases of H5N1 in wild birds reported from 5 December 2022 to 5 April 2023 by the Chilean National Animal Health Authority (21, 22) to the World Animal Health Information System (WAHIS) platform (4). These cases were tested by RT-qPCR using the VetMAX-Gold AIV Detection Kit (Applied Biosystems/Thermo Fisher Scientific 4,485,261), which targets the avian influenza M gene. Positive samples were confirmed by a specific H5 RT-qPCR assay (20). The samples were obtained from passive and active surveillance that included live and dead wild birds, backyard production birds, and industrial poultry. Sampling typically consisted of oral, tracheal, or cloacal swabs, but also included tissues when available from dead animals (20). From each record, we obtained species, date, geographical coordinates, condition (dead or alive), and whether the animals were killed or disposed. An outbreak was considered one entered line on the WAHIS system, and the total number of positive/suspected cases was considered in the number of cases per outbreak.

### 2.2 H5N1 spatio-temporal cluster analysis

Visualization of H5N1 outbreak locations was generated using QuantumGIS v.3.3 (23) and projected for analysis using the WGS 1984 datum as the coordinate system. Each sampled site was considered an H5N1 outbreak if at least one individual swab sample tested positive, according to the results of the specific RT-qPCR. First, spatial distribution was characterized by the global Moran’s I index (24), to identify spatial autocorrelation among the data. Then, we used the scan statistic (25) to detect any cluster of H5N1 outbreaks under space–time permutation probability modeling. The model was run using H5N1 locations under the null hypothesis that outbreaks were randomly distributed across the country. The model was set to scan for areas with high H5N1-positive numbers to test for clusters with a spatial occurrence higher than that outside the cluster. The model allowed the detection of the most “unusual” excess of observed H5N1 outbreaks and therefore provided georeferenced high-risk areas of H5N1 occurrence. Likelihood ratio distributions were obtained using the Monte Carlo simulation by generating 999 replications of the data set under the null hypothesis of a random distribution of cases in time and space (26). The analysis was performed using SatScan v.10.1 software, considering a *p*-value of <0.05 as statistically significant (27).

### 2.3 Ecological and anthropogenic H5N1 drivers

We first tested the association between distance and days since the first outbreak through a linear regression model. We also tested the correlation between the presence or absence of H5N1 outbreaks in wild birds and several ecological and anthropogenic variables that could be related to disease occurrence or reporting. For this purpose, we built a global model including all 19 bioclimatic variables from WorldClim at a spatial resolution of 30-s (~1 km^2^) (28), after checking that there were no major discontinuities with these variables in our study area (29). We also included a variable of bird richness obtained from the International Union for Conservation of Nature’s (IUCN) Red List of Threatened Species (30). To test whether H5N1 was more likely to occur in areas with high human presence or activity, our model included one of the three variables considered proxies of human activity: the human footprint as cumulative human pressure on the environment (31), human density, or total human population obtained from the global high-resolution population denominators project (32). Finally, the distance of the outbreak to the nearest urban center and the nearest SAG office were included as proxies of reporting effort, as distance to the reporting office may be negatively correlated with the probability of reporting (33). We extracted all data for each H5N1 outbreak to coordinates using raster layers of 30 s (~1 km^2^) spatial resolution with QuantumGIS v.3.3. Supplementary Table S1 details each assessed variable and data source.

We built a generalized linear model (GLM) with a binomial error structure (link = logit) to test the association between the H5N1 presence/absence model and the variables described above using the multGLM function in the *fuzzySim* R 4.2.1package. This function allowed us to previously test correlations between variables (particularly bioclimatic variables) and exclude them from the analyses to avoid collinearity problems. The model was set to test correlations using a rho >0.8 and a significance of *p*-value <0.05, retaining the variable that was more informative to the response variable. The reporting data for H5N1 only included H5N1 “presence” data, and no reports of H5N1 “absence” were available (e.g., reports of healthy birds that tested negative for H5N1). Thus, in order to include H5N1 “absence” points in our GLM binomial model, we created “pseudo-absence” points, i.e., locations where H5N1 was not reported, and from which all environmental/human variables used in the model were extracted. Pseudo-absence points were randomly selected within a 50-km radius buffer from the coordinates of reported outbreaks, which we assumed was a realistic radius of movement for wild bird species. From each pseudo-absence location, we extracted all the data above for H5N1 outbreaks. We selected a total of 445 points, representing 1 randomly selected point per outbreak location within a 50-km radius buffer. We ran the full model with all variables, excluding correlated variables according to the model (bioclim variables bio1, bio4, bio5, bio6, bio8, bio9, bio10, bio11, bio12, bio13, bio14, bio15, bio16, and bio17) and used the dredge function from the MuMIn package to select the model with the lowest AICc. Independent models using similar variables (e.g., three variables of human activities) were tested independently, and the model was selected based on its AIC. Model fit was evaluated using the *DHARMa* package (*testOutliers, testDispersion*, and *qqplot* functions) in R. The significance of spatial autocorrelation in the residuals of the full model was tested using Moran’s *I* test (34) in the *spdep* package, and no significant autocorrelation was found.

## 3 Results

### 3.1 H5N1 occurrence and species affected

**Figure 1:**
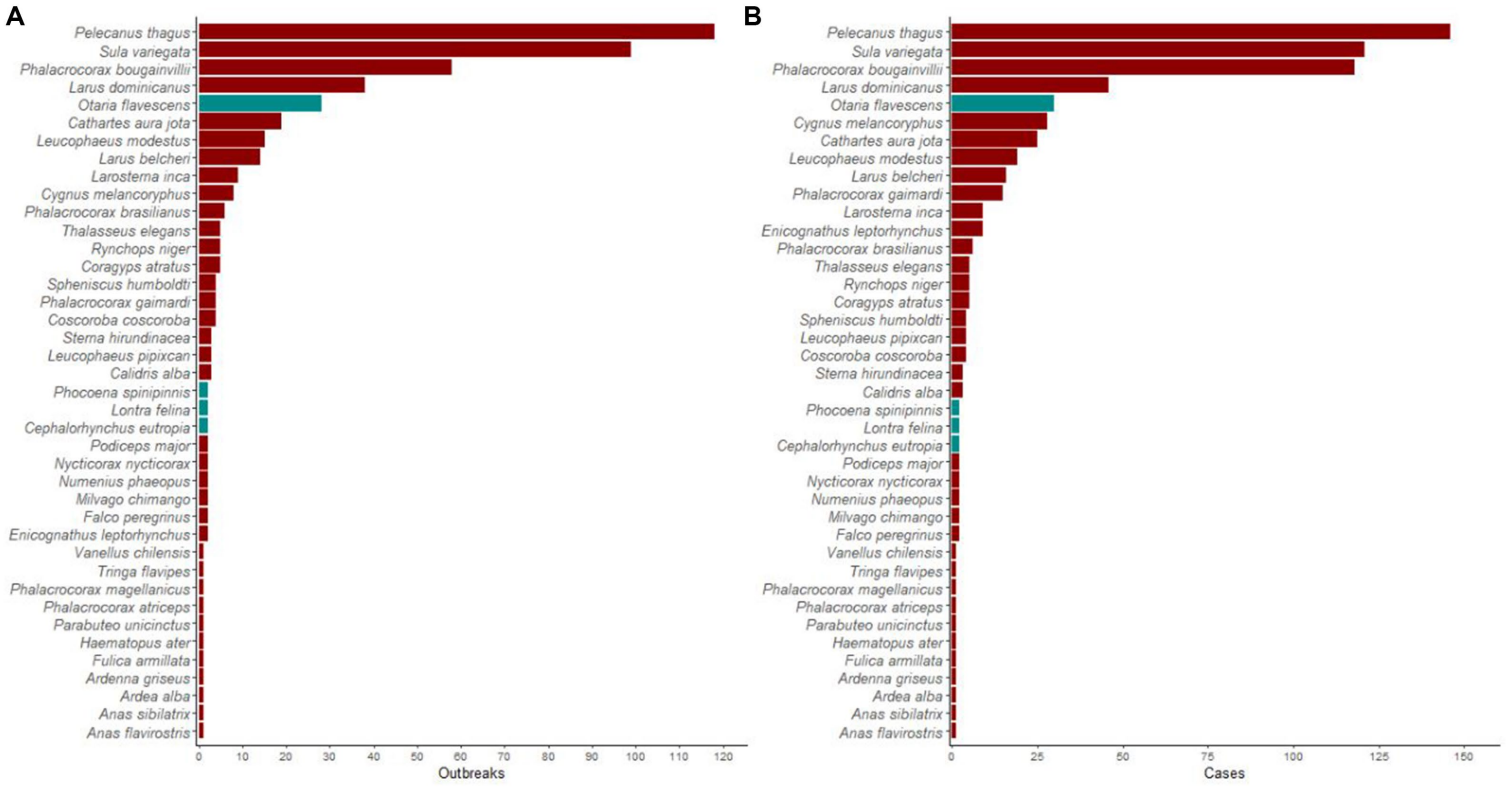
Highly pathogenic avian influenza H5N1 occurrence in wildlife in Chile. Histogram showing a number of **(A)** outbreaks and **(B)** individual cases of highly pathogenic avian influenza H5N1 in wild birds (red bars) and marine mammals (gray bars) in Chile between December 2022 and April 2023.

A total of 445 outbreaks, involving 613 individual wild birds of 36 species, tested positive for H5N1 in Chile. Most outbreaks involved *P. thagus* (*n* = 118, 27%), followed by Peruvian boobies (*Sula* var*iegata*; 99, 22%), guanay cormorants (*Phalacrocorax bougainvillea*; 58, 13%), and kelp gulls (*Larus dominicanus*; 39, 9%). Among marine mammals, *O. flavescens* predominated, with 28 reported outbreaks (not included in the analyses). Affected threatened species included the endangered Humboldt penguins (*Spheniscus humboldti*) and *L. felina*, with four and two outbreaks, respectively. Details of reported H5N1 outbreaks and individual cases among wild birds and marine mammals are presented in Figure 1.

### 3.2 H5N1 spatio-temporal cluster analysis

**Figure 2:**
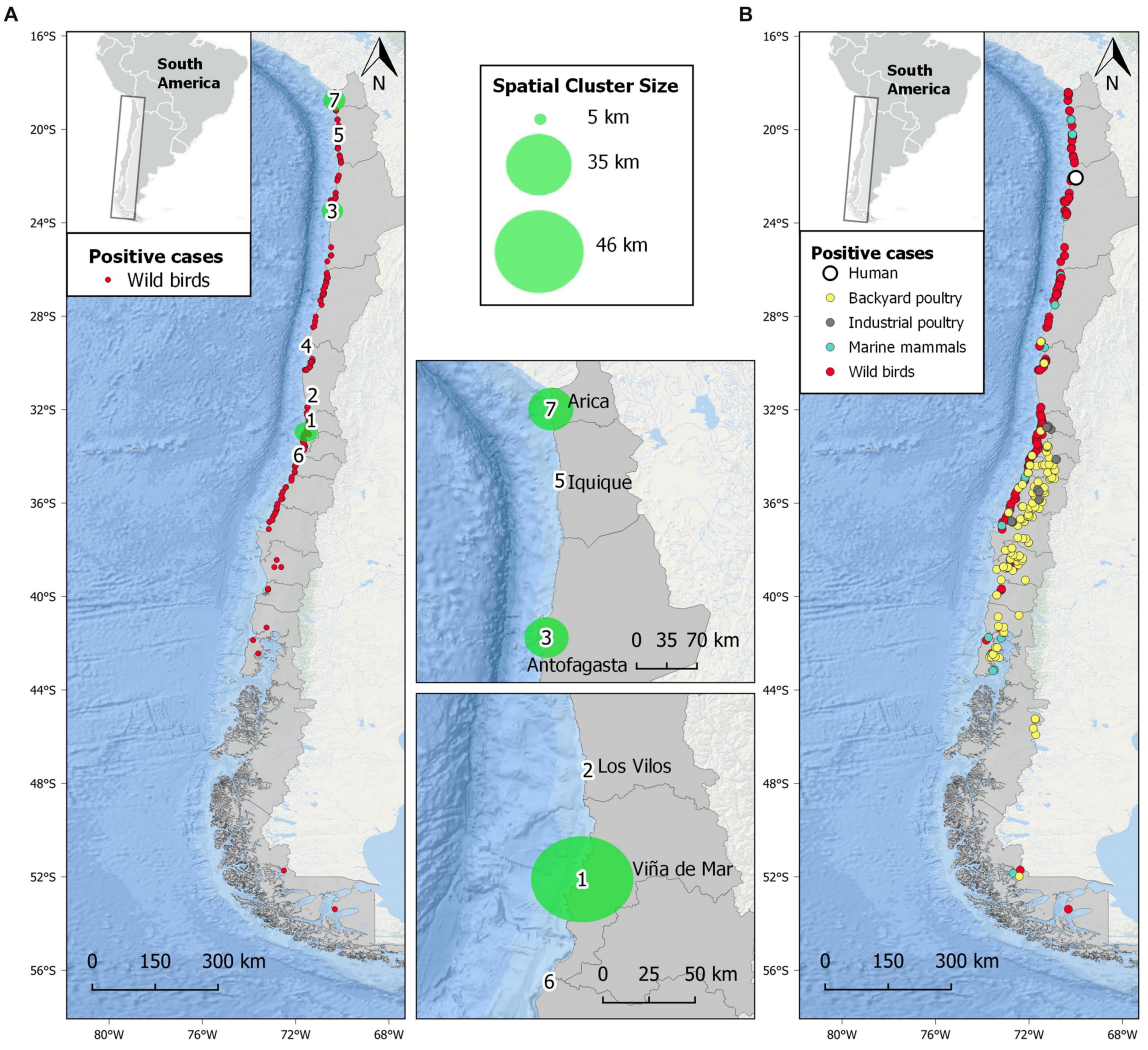
Spatial distribution of highly pathogenic avian influenza H5N1 in Chile studied from December 2022 to April 2023. **(A)** Distribution of H5N1 outbreaks in wild birds and seven statistically significant (*p* < 0.05) spatial clusters of H5N1 high-risk areas obtained from scan statistics analysis. Larger clusters are represented by green-shaded circles and are detailed in the insets. **(B)** Distribution of all confirmed outbreaks of H5N1 in Chile, including wild birds, marine mammals, backyard and industrial poultry, and a human.

Our space–time permutation analysis detected seven statistically significant clusters of H5N1 outbreaks, following a pattern in which the progression to the south of the H5N1 epidemic wave can be observed (Figure 2 and Supplementary Table S2). Space–time clusters were scattered from the extreme north to central Chile and ranged from 6 to 17 individual H5N1-positive birds in each. One of the clusters had a radius of <1 km, while the remaining six had a variable radius of 2 to 49 km. Three clusters (#1, 2, and 6) were located in central Chile, close to the most densely populated area in the country. The largest clusters had a radius of >45 km and were located in central (#1) and north Chile (#3 and 7). The city of Tocopilla is situated between two nearby clusters (#3 and 5), and it is the location where the first human case of H5N1 was reported later (Figure 2B). The other two initial cases in the backyard and industrial poultry were also predicted by clusters (#4 and 6, respectively). The Global Moran’s *I* index was statistically non-significant (*p* = 0.2), indicating that there is no spatial autocorrelation of the data.

### 3.3 Ecological and anthropogenic H5N1 drivers

**Figure 3:**
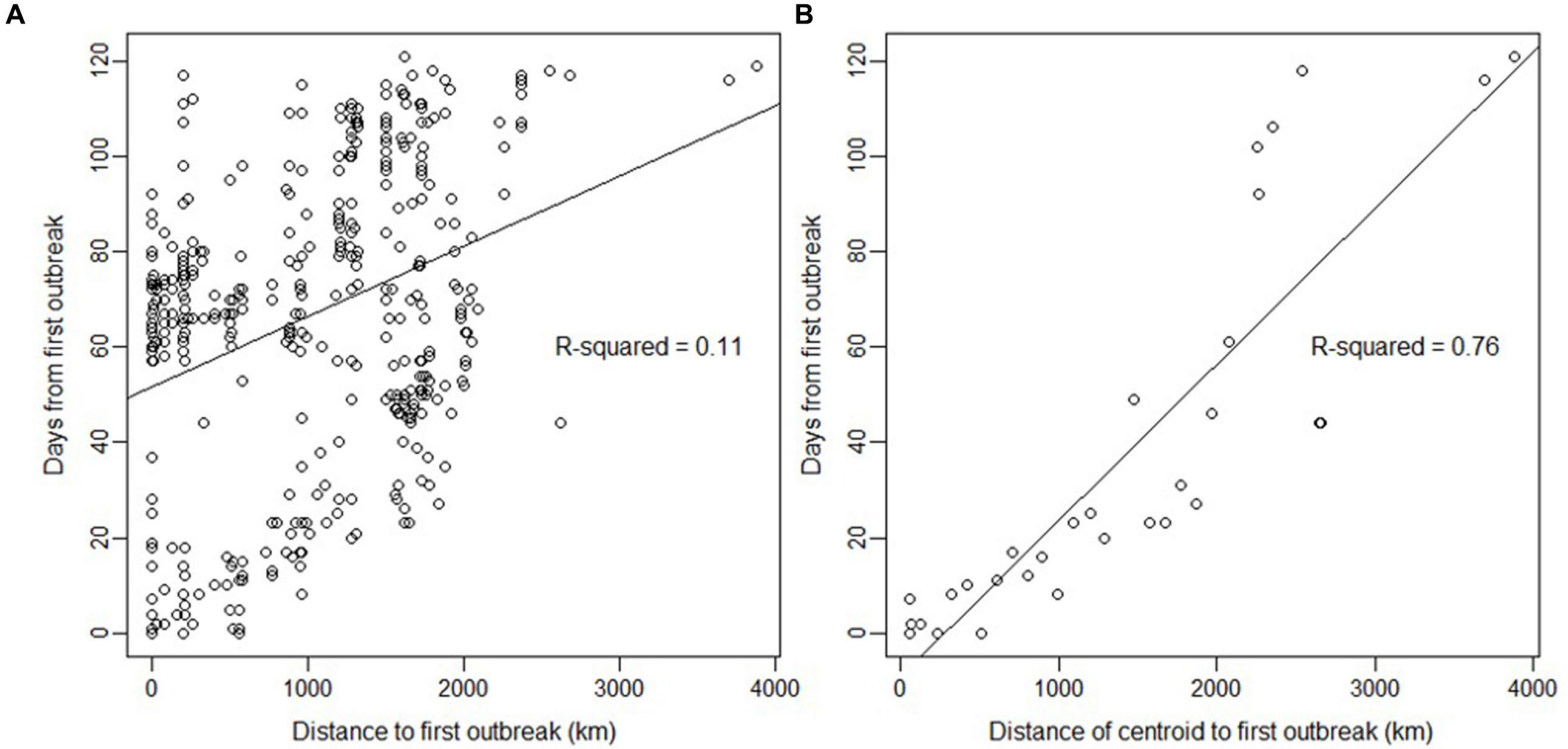
Relationship between days and distance since the first outbreak of highly pathogenic avian influenza H5N1 in wild birds in Chile. **(A)** Relationship using the coordinates of each outbreak (*n* = 445). The lines are predictions from the linear model (*R*-squared is given for the prediction). **(B)** Same analysis but using the distance from centroids and the date of the first case detected on the centroid (*n* = 31), with a slope of 32.76 days/1000 km. Centroids were estimated using a cell size grid = 1 with the *st_make_grid* function of the sf package in R. The northernmost outbreak of the first day of reporting in the dataset was used as the first index case for all calculations.

**Figure 4:**
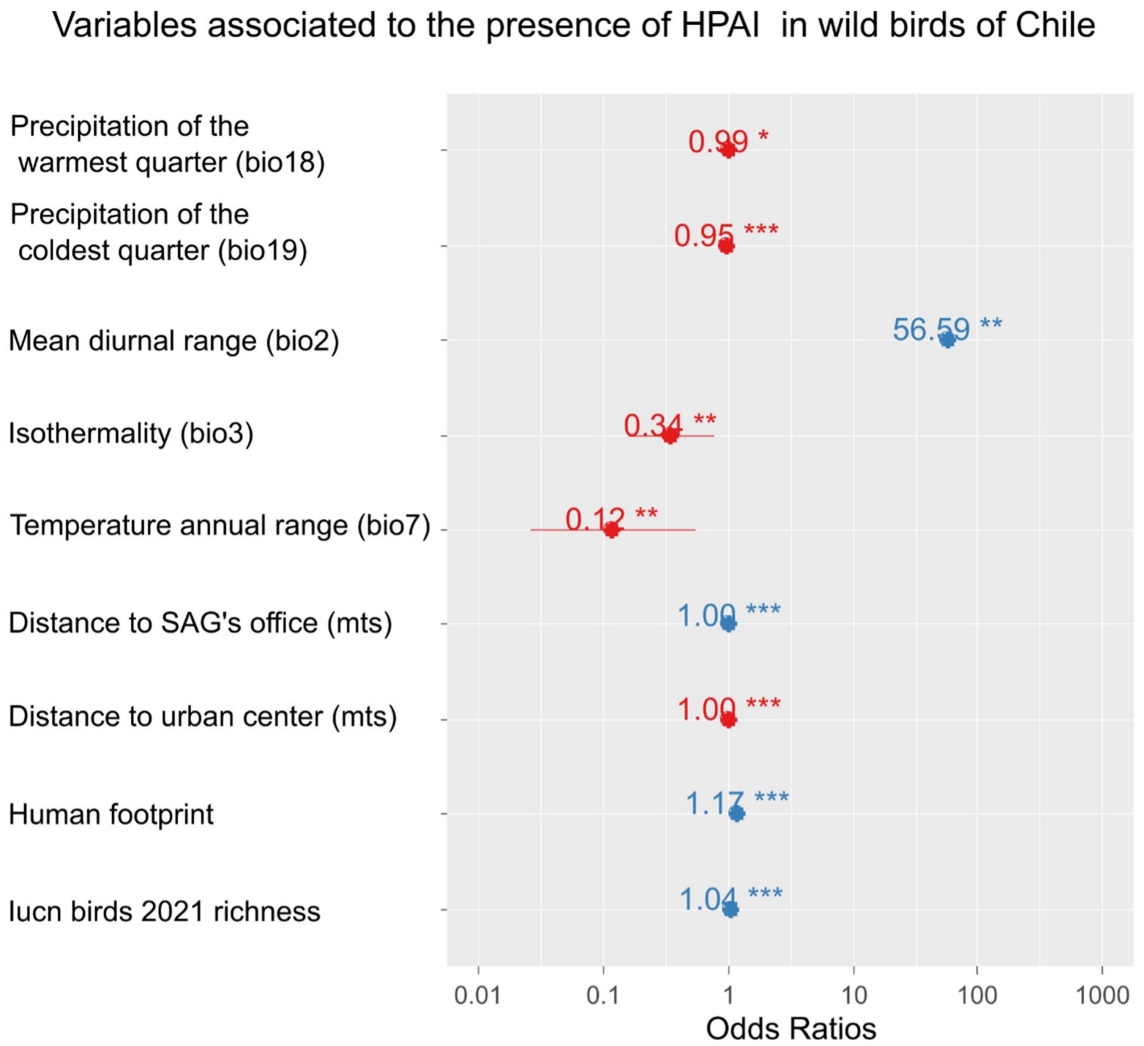
Ecological and anthropogenic variables associated with the presence of highly pathogenic avian influenza H5N1 outbreaks in wild birds in Chile. Effect size (odds ratio) of variables explaining the presence/absence of cases using a GLM with binomial error distribution. Odds ratios with an asterisk show statistically significant effects (*p*-value <0.05); the more asterisks, the lower the *p*-value.

A strong linear relationship was found between time and distance from the first outbreak (Figure 3), suggesting a wave-like steady spread from north to south. The linear relationship was high (*R*^2^ = 0.76) when using the grid’s centroids. Furthermore, the presence of H5N1 outbreaks in birds was correlated with several ecological and human-related variables. The presence of H5N1 was positively correlated with bird richness, human footprint (also population density or human total population, but with a lower model fit), distance to SAG’s office, and mean diurnal range (bio2; GLM, *p* < 0.05). In contrast, the presence of H5N1 was negatively correlated with the distance to the closest urban center, isothermality (bio3), temperature annual range (bio7), precipitation of the warmest quarter (bio18), and coldest quarter (bio19; GLM, p < 0.05, Figure 4 and Supplementary Table S3). No significant association was found between the presence of H5N1 and other bioclimatic variables (GLM, *p* > 0.05).

## 4 Discussion

Since its original description in southern China in 1996 (35), H5N1 has caused its highest impacts and has become more widespread than ever, heavily impacting wildlife and domestic birds across the Americas since late 2021 (1, 7, 14, 16, 36, 37). However, its spatio-temporal dynamics, the epidemiological role of affected hosts, and factors associated with outbreaks remain poorly understood. In this study, we described the dynamics of the outbreaks reported in Chile, showing several clusters across the country, a wave-like spread from north to south likely generated by the arrival of migratory birds in the spring, and several ecological and human variables associated with outbreaks, from which recommendations for disease management and future research are provided.

Outbreaks involving highly pathogenic avian influenza H5 viruses in domestic birds have been well-known in Europe, North America, Asia, and Africa over the last century, causing vast economic losses (7). Since 1996, H5N1 has been reassorting with low pathogenic avian influenza viruses circulating in both domestic and wild bird populations, first in Southeast Asia, but since 2005, spreading to the rest of Asia, Europe, and Africa through migratory birds (38). In these regions, H5N1 has caused recurrent mortalities among poultry, wild birds, and humans, highlighting the significance of continued surveillance and understanding of the epidemiology of this highly lethal disease (5).

Our analyses show that the novel H5N1 virus has spread fast across Chile, causing high mortality in a diverse range of species. In 4 months, the virus spread 4,000 km along the Pacific flyway in Chile, with infection to date being reported in 51 wild birds, 4 domestic birds, and 5 marine mammal species (21, 22). Unprecedented mortalities of over 100,000 wild animals (80,000 wild birds and 20,000 marine mammals) have been documented in Chile since December 2022 (21, 22). This epidemic pattern is similar to the one observed in North America, where the virus spread rapidly throughout the continental United States and all provinces of Canada, affecting more than 28,000 wild birds based on disease and mortality estimates (39). A similar situation occurred in Peru, where more than 200,000 wild birds (37) and 5,200 marine mammals (14) have died since October 2022. In addition, in the United States, H5N1 has been associated with high mortality rates and breeding failure in the bald eagle (*Haliaeetus leucocephalus*) and with unusual mortalities in the highly threatened California condor (*Gymnogyps californianus*), thus far killing 6% of the wild population (40). Likewise, the arrival of H5N1 to Tierra del Fuego in the extreme south of Chile is currently an imminent threat to Antarctic fauna. In continental Chile alone, >3,000 *S. humboldti* have died from suspected H5N1 since January 2023 (22). There is no information available on why this species appears to be so susceptible to the virus.

Genetic sequencing of Chilean H5N1 samples indicates that they all cluster together within the clade 2.3.4.4b (20, 41, 42), suggesting that the outbreaks in the country all belong to the same viral lineage (with potentially minor variations) that is globally widespread. This virus arrived in Chile from neighboring Peru, and previously from North America (2, 36). This wave-like spread is likely associated with the movement of wild birds, although no information is currently available on the host species disseminating the virus across space in South America. Although pelicans, boobies, gulls, and cormorants have been the most affected species in Chile and Peru (1, 37), ducks and teals are recognized as reservoirs and spreaders of avian influenza in other regions (43–45) and given that the movement behavior of most of these species lacks large-scale migrations, it is unknown which species are responsible for the observed pattern of spread in Chile (46, 47). Thus, further research combining spatial ecology and genetic analyses could help solve this relevant question.

Studies have shown that the black-headed gull (*L. ridibundus*) in Europe is highly susceptible to avian influenza, likely contributing to local transmission rather than long-distance spread due to their high mortality to H5N1 (48). Gulls are among the bird species most involved in outbreaks in Chile. The largely sedentary behavior and high-density aggregations in colonies during breeding exhibited by *L. dominicanus* and Belcher’s gull (*L. belcheri*) suggest that these species may act as competent H5N1 reservoirs, contributing to virus persistence in areas such as our identified clusters. The situation is different for the gray gull (*L. modestus*), as its migratory condition could help spread H5N1 along much of the Pacific flyway within South America (49). Due to their scavenging habits and migratory behavior in the Americas, turkey vultures (*Cathartes aura*) could also contribute to the spread of H5N1 (37). Conversely, Anseriformes were involved in few outbreaks in Chile, despite being recognized as key hosts in the epidemiology of avian influenza viruses, both as reservoirs (40, 45, 50–52) or as long-distance spreaders (53). As previously shown for a wide variety of low pathogenic avian influenza viruses in Chile (52, 54), it is possible that as H5N1 becomes ezootic, and with Anseriformes start playing more relevant epidemiological roles, such as acting as virus reservoirs (6, 44) or even suffering the consequences of disease (53). For instance, the unusual mortality of approximately 800 black-necked swans (*Cygnus melancoryphus*) in a wetland in Valdivia (southern Chile) associated with H5N1 is of high concern (21).

Our permutation space–time model was useful in identifying high-risk areas of H5N1 outbreaks, guiding efforts to focus disease monitoring and carcass removal. To date, 12 outbreaks have been reported in industrial poultry units and 175 in backyard poultry production systems in Chile, in which over 1,000,000 birds have died or been killed for disease control management (21). This is due to the high industrialization of commercial poultry production in Chile, which has high biosecurity standards, compared to backyard poultry production, which is generally developed in open areas where domestic and wild birds share the environment, thus facilitating disease transmission (55). Our spatio-temporal analyses showed that clusters occurred most frequently close to urban centers, confirming our modeling approach showing a correlation between H5N1 occurrence and human-populated areas. For example, central Chile is a major hub for chicken production in the country, where three (of seven) H5N1 clusters were identified. The location of large cities with high demand for poultry products would increase the chances of disease transmission and spread through road networks (56–59). The observed close distance from outbreaks to highway junctions (e.g., Pan-American Highway; < 30 km) is worrying since highway junctions have been considered “dissemination nodes” for influenza viruses in poultry (56–58). Industry production and trade close to urban and rural wetlands (e.g., Aconcagua River mouth) can increase the chances of infection because of the higher risk of contact between domestic waterfowl and wild birds (60). Ongoing spillover from wild birds to poultry also threatens Latin America’s food security, as the region includes some of the world’s largest poultry producers, such as Brazil, Mexico, Colombia, and Argentina (61).

The wave-like spread south of the novel H5N1 in Chile allows for the prediction of new outbreaks. This can help in implementing preventive measures in farmed birds, other domestic animals, and people, or speed up mitigation actions in wild populations. For example, regular wave-like patterns of vampire bat rabies in Peru have contributed to alerting communities ahead of the spreading wave to implement preventive strategies such as vaccination (62). We also showed that the outbreak’s occurrence in wild birds was associated with several bioclimatic variables and proxies of human activities. For example, in our study, H5N1 occurrence was negatively associated with distance to urban centers and positively associated with human footprint, likely due to a lower reporting capacity and detection probability as distance from cities increases. For instance, H5N1 RT-qPCR testing in Chile is centralized in the SAG’s virology laboratory in Santiago, possibly contributing to a geographic detection bias. In fact, the same pattern did not occur when SAG offices were analyzed. In contrast, a significant positive correlation was found between H5N1 occurrence and the distance to the nearest SAG office. SAG offices are distributed along the country with a strong presence where agriculture and livestock develop, which can coincide or not with human-populated areas. For instance, many offices are located inland (including border crossings in the mountains) or in Patagonia, far from the described H5N1 outbreaks. In addition, bird species richness was positively associated with outbreaks in our study, suggesting that areas of high bird diversity can be hotspots of H5N1, as previously found for bird richness and prevalence of low pathogenic avian influenza viruses in Spanish wetlands (63). Additionally, Huang et al. (64) found that bird species richness, particularly of higher-risk species (i.e., *Larus* spp.), showed a positive relationship with H5N1 outbreak probabilities in wild birds in Europe. Among climatic variables, the mean diurnal range had the highest positive association with outbreaks’ occurrence (~57 times per temperature degree), while other variables including isothermality, temperature annual range, and precipitation of the warmest quarter and the coldest quarter showed a negative, but smaller, association with H5N1. These findings encourage research on ecological modeling predictions of future outbreaks, particularly for the upcoming critical winter period, which has shown a peak of H5N1 outbreaks in North America (39) and Europe (6, 65). Similarly, in a study in central Chilean wetlands based on fecal samples of wild birds, Ruiz et al. (54) found a positive association between the prevalence of low pathogenic avian influenza viruses and the minimum temperature for the month of sampling. Although previous studies in Chile have found a higher prevalence of enzootic avian influenza viruses during summer and autumn (52, 54), the current progression of the H5N1 epizootic in South America prevents testing for seasonality effects. Furthermore, as the epizootic moves inland, it is important to include in future studies the analysis of other ecological variables that might be relevant, such as vegetation coverage, the size of water bodies (54) and host density (50). The biological mechanisms behind the observed associations found in our study with several bioclimatic variables in terms of host and viral dynamics open venues for future research.

One of the most worrying issues of the current panzootic is the increasing cases of H5N1 infections in wild mammals, both marine and terrestrial, causing unprecedented mass mortalities (3, 13–15, 36). The current epizootic associated with mortality in *O. flavescens* and *L. felina*, among other marine mammals, is the first report of H5N1 in these species. Worryingly, RNA sequencing has shown signs of early adaptation of the H5N1 2.3.4.4b clade virus to wild mammals (66) and humans (41), fueling fears of a new human pandemic (18). In addition, recent reports of a dog and two cats dying from H5N1 after contact with dead birds in North America (67) raise the potential of wildlife-to-dog transmission in South American countries such as Chile, where a high population of dogs (both owned and stray) occurs (68). Furthermore, additional lethal cases in domestic cats have been recently reported (69, 70). Even more, two cases of zoonotic avian influenza have recently been reported in the region [Ecuador and Chile; (41, 71)], with the Chilean case being of special concern. Here, the location of this case (Tocopilla) was predicted as a high-risk area with high precision by an earlier spatio-temporal permutation analysis. Later, by adding more (scattered) data, this case falls between two clusters in northern Chile (#3 and 5, Figure 2). The epidemiological investigation concluded that the transmission most likely occurred through environmental exposure due to the high number of dead sea lions and wild birds near the infected man’s residence (41, 72), showing the potential of H5N1 spillover to humans from wildlife even in the absence of direct contact.

## 5 Conclusion

Our results identified a wave-like steady spreading of the novel H5N1 virus in Chile. We predicted hotspots of H5N1 risk and factors associated with outbreak occurrence, contributing to establishing targeted prevention measures to avoid further spread and spillover to other species, including humans. To date, the H5N1 epidemic has caused the death of more than 100,000 wild animals in Chile, so mitigation strategies are urgently needed to reduce the impact of this emerging disease on wildlife, including endangered species such as penguins (e.g., *S. humboldti*), and the imminent arrival of the virus in Antarctica. Traditionally, strategies to control avian influenza outbreaks have focused on poultry, but new and urgent efforts are needed to contain virus circulation in wild populations and the environment. Preventive actions based on our modeling approach include developing wildlife surveillance diagnostic capabilities with a focus on our predicted H5N1 high-risk areas identified by our spatio-temporal cluster analysis and general linear modeling (i.e., areas with a higher temperature, bird richness, and human footprint). It is also necessary for scientists, local communities, the poultry sector, and national health authorities to co-design and implement science-based measures from a One Health perspective to avoid further spillover to domestic animals and humans. Mitigation of H5N1 in wild populations remains a major challenge. Rapid removal and proper disposal of dead Sandwich terns (*Thalasseus sandvicensis*) in north-western European colonies suffering high impacts of H5N1 reduced mortality by 15% (73). Furthermore, the closure of public areas (i.e., beaches) reporting high wildlife mortalities appears to be reasonable to decrease the risk of zoonotic transmission. Likewise, target surveillance of those key wild species identified in our study, including bird banding, wetland monitoring, and citizen science, could all contribute to a better understanding of the epidemiology of H5N1. Increasing wildlife disease surveillance and genomic sequencing is also essential to better understanding cross-species transmission rates and how the virus is potentially adapting to mammals. Our study highlights the urgent need for further research to identify, alert, and prevent hotspots of H5N1 transmission, limiting the potential for a new pandemic emergence, a food security catastrophe, and further biodiversity loss.

## Data availability statement

Data used for modeling of the 613 individual RT-qPCR assays in wild birds for H5N1 detection is available in the Supplementary material.

## Ethics statement

Ethical approval was not required for the study involving animals in accordance with local legislation and institutional requirements because we used a database and did not perform any sampling or manipulation of animals.

## Author contributions

CA: Conceptualization, Funding acquisition, Investigation, Methodology, Project administration, Resources, Supervision, Validation, Writing – original draft, Writing – review & editing. MA-R: Formal analysis, Methodology, Visualization, Writing – original draft, Writing – review & editing. JA: Data curation, Formal analysis, Methodology, Software, Writing – original draft, Writing – review & editing. JB: Conceptualization, Formal analysis, Investigation, Methodology, Project administration, Software, Writing – original draft, Writing – review & editing.
